# Programmable metasurface for front-back scattering communication

**DOI:** 10.1515/nanoph-2023-0365

**Published:** 2023-08-03

**Authors:** Haipeng Li, Kewei Xin, Haiyang Ding, Tangjing Li, Guangwei Hu, He-Xiu Xu

**Affiliations:** National University of Defense Technology, Test Center, 710106, Xi’an, China; National University of Defense Technology, College of Information and Communication, 430035, Wuhan, China; Air Force Engineering University, Air and Missile Defense College, 710051, Xi’an, China; Nanyang Technological University, School of Electrical and Electronic Engineering, 637371, Singapore, Singapore

**Keywords:** amplitude reconfigurability, front-back scattering communication, polarization conversion, programmable metasurface

## Abstract

Achieving high-efficient and low-power communication is pivotal yet very challenging in the emerging technologies. Unlike conventional backscatter communication system, we propose and demonstrate an amplitude-reconfigurable metasurface loaded with PIN diodes to build a front-back scattering communication transmitter, which features the exclusive advantages of full-space secondary modulation of the ambient signals with high energy utilization efficiency. Meanwhile, this device can eliminate the interference originated from the ambient source by polarization conversion in the transmission channel. At a modulation rate of 800 kbps and a distance of 80 m, our system can achieve distortion-free transmission of a picture with size of 200 × 200 pixels. In addition, multiple amplitude-shift-keying modulation is also realized by segmenting the metasurface to further increase the communication rate. Due to the advantages of high spectral efficiency and low energy consumption, this system can be widely used in future engineering applications for the internet of things, especially for smart home, agriculture environmental monitoring, wearable sensing and others.

## 1 Introduction

Nowadays, the internet of things (IoT) has been revolutionizing all aspects of our life, from smart city infrastructures, smart home, *ad hoc* healthcare and various others. With the advent of the 5G era featuring unprecedented communication speed and capability, a grand vision of the internet of everything has been chased, where several important challenges however remain to be solved such as the lack of spectrum resources, high energy consumption, and high maintenance cost among others [1]. Primarily, reducing the power consumption of powerful IoT devices, especially the communication module, is fundamental.

Currently, backscatter communications (BC) are mostly investigated as one key solution. BC systems modulate the incident electromagnetic (EM) waves (as the carrier source) and transform the information via scattering them with the matching state of the antenna [2–4]. Here, no active carrier source is required, thus avoiding the energy-drawing devices (such as oscillators and power amplifiers) and approaching the microwatt practical power consumption. There are three main types, depending on the source of the carrier wave: the single-station, dual-station and ambient BC systems. Thereinto, ambient BC, firstly proposed and implemented in two passive devices in 2013 [2], is an emerging technology that exploits carrier source such as TV or WiFi signals, which are abundant particularly in IoTs. As a result, ambient BC system attracts the widespread attention in IoTs, for their advantages of low energy consumption, low cost and high spectral efficiency. However, the interference of ambient signals becomes the major concern. To eliminate the signal crosstalk, dual antenna receivers are proposed to enhance the distance of BC [3], which, as a side effect, complicates the receiver design and detection [4–7].

Programmable metasurface may offer the solution to solve this issue of ambient BCs. it can reconfigure the characteristics of EM waves on an ultra-thin, low-loss and easily fabricated deeply subwavelength structures such as printed-circuit-board (PCB) in the microwave region. To date, various exciting applications of metasurfaces have been demonstrated, such as anomalous refraction/reflection [8–10], beamforming [11], propagating wave to surface wave transformation [12, 13], multi-beam wave generation [14], radio-frequency energy harvesting [15–17], cloaking [18–21], circular polarization wavefront manipulation [22, 23], meta-lens [24], space-time modulation [25–27], wireless communication [28–30], intelligent indoor robotics [31], and some others [32–34]. Furthermore, the use of reconfigurable metasurfaces to achieve BC systems has attracted great interest due to their natural integration on what regards [35]. Regarding the concept of BC and metasurface, the systems can be recategorized as three major types: non-modulated-metasurface BC, modulated-metasurface BC, and ambient modulated-metasurface BC [36]. In a variety of metasurfaces, the omni-directional metasurface, which can simultaneously steer the reflected and transmitted waves, has more advantages over the traditional BC antennas.

**Figure 1:**
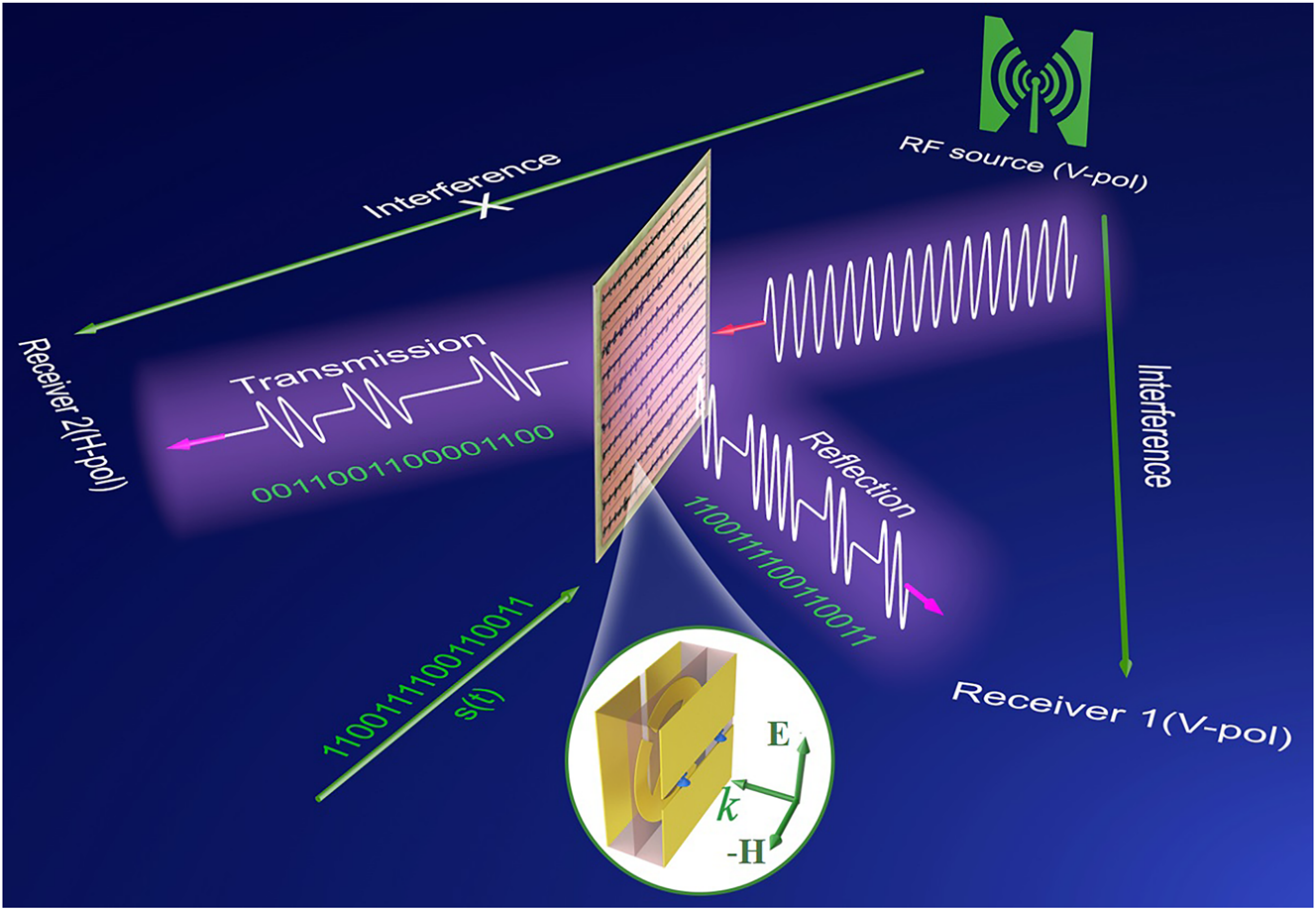
Schematic function of the programmable metasurface for front-back scattering communication transmitter. When PIN diodes on the metasurface are on, the carrier wave (gray sine wave) will be reflected and the reflection signal exhibits the same polarization as the carrier wave. When PIN diodes are off, the carrier wave will be transmitted and the transmission signal manifests orthogonal polarization so that the interference from the RF source will be eliminated. In addition, the reflection and transmission signals are modulated according to the input control signal *s*(t) of 1100111100110011.

Here, a programmable metasurface-based front-back scattering transmitter is proposed and demonstrated (see Figure 1). Such an omni-directional metasurface is loaded with and controlled by positive-intrinsic-negative (PIN) diodes: when the diodes are turned on, the incident vertical-polarization (V-pol) waves will be fully reflected; while off, the incident V-pol will be mostly transmitted along with a polarization conversion to a horizontal polarization (H-pol) to minimize the (polarized) signal crosstalk. Hence, the on-off keying (OOK) modulation of the data can be achieved. Due to the complementary nature of reflection and transmission power, the metasurface-based transmitter can transfer data to both forward and backward directions. Compared with traditional BC transmitters, this work not only improves the energy utilization efficiency greatly by using total energy for communication, but also provides effective method to eliminate the direct interference. Meanwhile, multiple amplitude-shift-keying modulation can be realized by segmenting the metasurface, which may further increase the communication capability. As a proof-of-technology demonstration, we showed a metasurface-based front-back communication transmitter near 2.45 GHz that can be used in WiFi-based smart home applications. We envision our proposed programmable metasurfaces as front-back scattering communications and may empower the IoT for their reconfigurable functionalities, low power consumption, low footprint, easy integration of existing devices, and other merits.

## 2 Concept of front-back scattering communication

**Figure 2:**
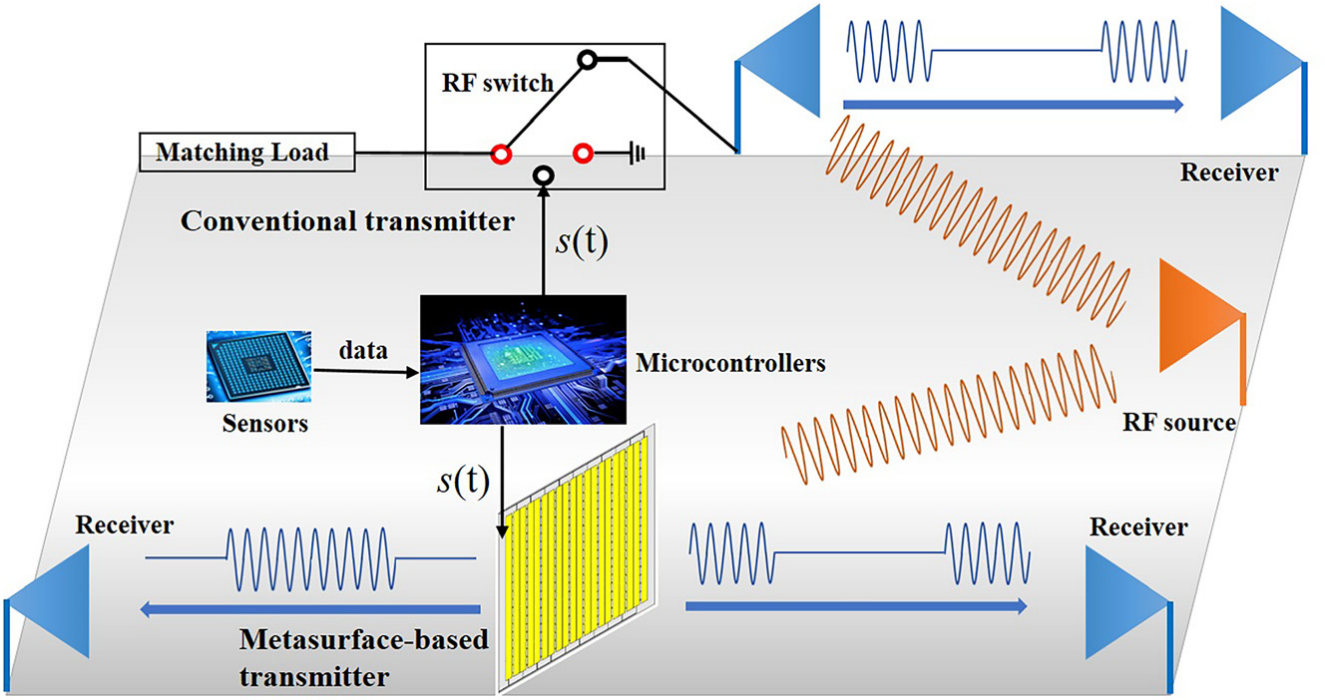
The concept and modulation strategy of the metasurface-based front-back scattering communication as well as conventional BC. For conventional transmitter, the RF carrier wave (orange sine wave) will be reflected to free space (blue sine wave) and absorbed by the matching load when the RF switch is off and on, respectively. For the metasurface-based front-back scattering communication transmitter here, the carrier wave (orange sine wave) will be reflected and transmitted when the PIN diodes are on and off, respectively.

The concept of metasurface-based front-back scattering communication is inspired by conventional BC, which indicates either ‘0’ or ‘1’ bit by switching its antenna between reflecting and non-reflecting states. For conventional BC transmitter, the reflecting and non-reflecting states are achieved by loading a radio-frequency (RF) switch in the feeding circuit (see Figure 2). When the switch is off (on), the antenna works at a mismatching (match) condition so that the receiving RF signals will be (not be) reflected to free space. The RF switch is usually controlled by a low-power microcontroller such as MSP430, and the control signal *s*(t) is generated according to the data returned by the sensors. Inspired by traditional transmitter, we proposed the concept of the programmable metasurface-based transmitter. As also shown in Figure 2, the reflecting and transmitting states are achieved by loading PIN diodes on a massive of meta-atoms. The incident signal will be reflected when the PIN diodes are turned on, while transmitted when the PIN diodes are turned off.

For conventional transmitter with “on” state, the energy has been absorbed by the matching load and not used for communication. While for the metasurface-based transmitter here, the full energy has been used for communications in reflection or transmission channels with the PIN diodes “on” or “off” state, respectively, which significantly improves the energy utilization efficiency. As shown in Figure 2, all receivers in front of and behind the metasurface can acquire valid signals from the transmitter. We call this kind of transmitter as front-back scattering communication transmitter to distinguish it from the traditional backscatter transmitter.

Meanwhile, the metasurface-based transmitter can enhance the communication distance by controlling the polarization and direction of the output RF signals. For OOK modulation of conventional backscatter transmitter, the received digital signal obtained by analog-to-digital conversion can be expressed as the following:

(1) $$y\left[n\right]=x\left[n\right]+\alpha B\left[n\right]x\left[n\right]+\omega \left[n\right]$$

where *x*[*n*] is digital sampling of RF carrier signal sources, *ω*[*n*] is additive noise of the receiver, *α* denotes complex attenuation coefficient of the backscatter signal, and *B*[*n*] = 0 or 1 denotes the bit stream of data emitted by the BC transmitter. By averaging *N* digital samplings of the receiver, we can obtain the average power of the received signal as following:

(2) $$\frac{1}{N}\overset{N}{\underset{n=1}{\sum }}{\left|y\left[n\right]\right|}^{2}=\frac{1}{N}\overset{N}{\underset{n=1}{\sum }}{\left|x\left[n\right]+\alpha B\left[n\right]x\left[n\right]+\omega \left[n\right]\right|}^{2}$$

Let $P=\frac{1}{N}\overset{N}{\underset{n=1}{\sum }}{\left|x\left[n\right]\right|}^{2}$ denotes the average power of the carrier signal. Assuming that the effect of noise is ignored, when *B*[*n*] = 1, the average power of the received signal is ${\left|1+\alpha \right|}^{2}P$ . While *B*[*n*] = 0, the average power of the received signal is *P*, which means that the carrier signal reaches the receiver directly. Due to this interference from the carrier signal, when *α* << 1 (the communication distance is long enough), ${\left|1+\alpha \right|}^{2}P$ and *P* are almost equal so that the states of 0 and 1 cannot be decoded correctly.

To solve this bottleneck, we devise a metasurface scheme with dynamic function of cross polarization conversion to eliminate the direct interference from the carrier signal. Due to the function of cross polarization conversion, the polarization of receiver should be orthogonal to that of the carrier signal so that the received digital signal can be rewritten as following:

(3) $$y\left[n\right]=\alpha B\left[n\right]x\left[n\right]+\omega \left[n\right]$$

where the term of direct interference (*x*[*n*]) is eliminated. In this condition, when *B*[*n*] = 1 and 0, the average power of the received signal is ${\left|\alpha \right|}^{2}P$ and 0, where the noise is also ignored. By determining the value of ${\left|\alpha \right|}^{2}P$ and 0, the bit of 0 and 1 can be decoded correctly, even *α* is quite small.

## 3 Design of front-back scattering communication transmitter

**Figure 3:**
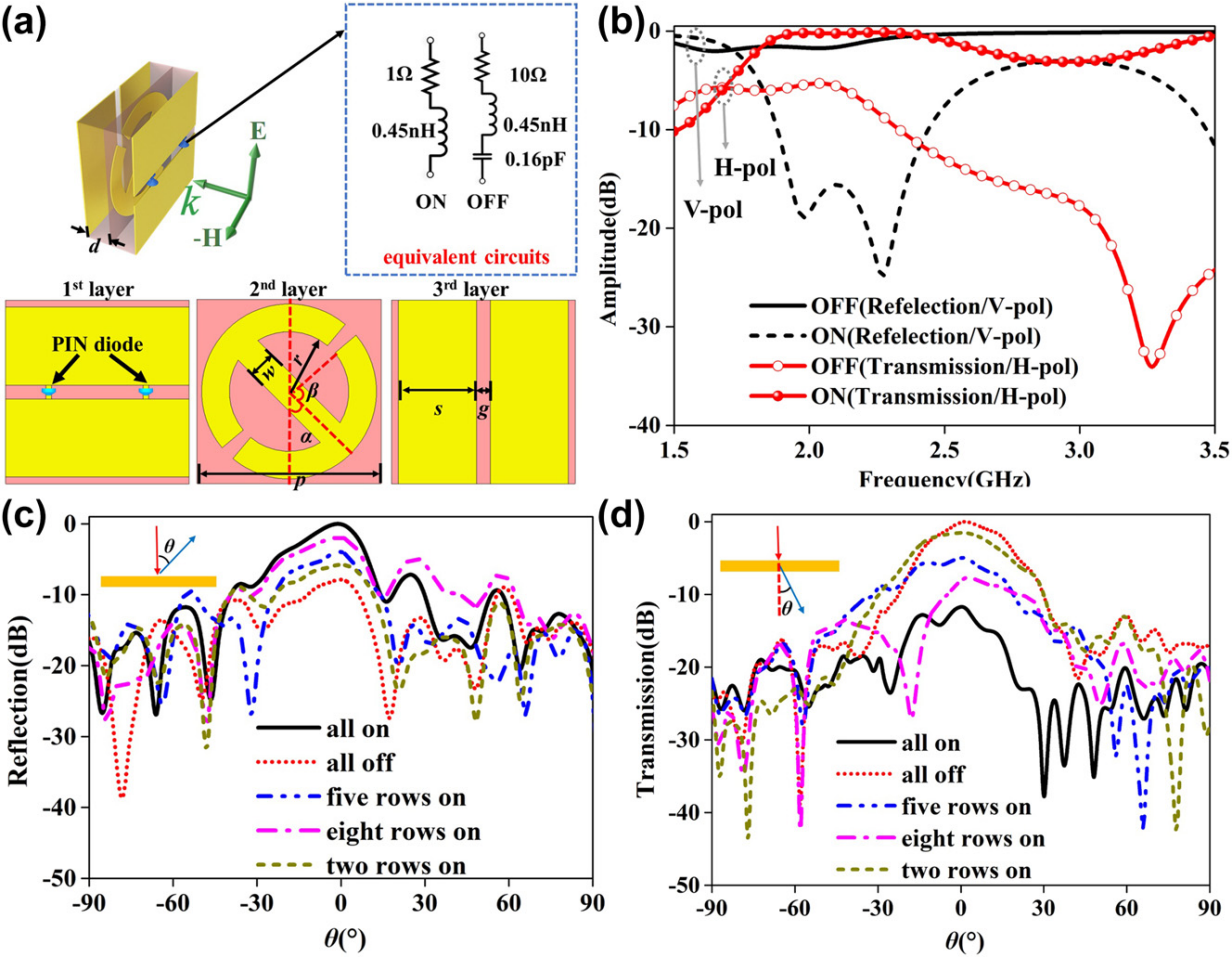
Characterization of the front-back scattering communication transmitter meta-atom. Three metallic layers are all printed on FR4 boards with thickness of 3 mm, dielectric constant (*ε*_*r*_) of 4.4. Geometric parameters are set as following, *d* = 3 mm, *p* = 20 mm, *w* = 3 mm, *r* = 9.5 mm, *α* = 45°, *β* = 88°, *s* = 8.5 mm, and *g* = 1.5 mm. (a) Structure of the meta-atom as well as the separate layers and equivalent circuit model of the PIN diode, here yellow part represents the metal and blue arrows indicate the PIN diodes. (b) The normalized reflection and transmission coefficients of the meta-atom with the PIN diodes being on and off. The far-field patterns for (c) V-pol reflection and (d) H-pol transmission at 2.45 GHz.

To realize the strategy proposed above, a dynamic meta-atom consisting of three metallic layers (marked as 1st, 2nd and 3rd layer) is devised and numerically studied as shown in Figure 3(a). Here, 1st and 3rd layers are orthogonal gratings while 2nd is a circular I-shape particle. According to our previous work [11], this kind of element without loading PIN diodes has high-efficiency cross-polarization transmission of EM waves over a wide frequency band. To achieve OOK modulation, the first layer of grating is loaded with two PIN diodes (Skyworks SMP1340-040LF). The equivalent circuits of PIN diodes with switchable on and off states [37] are shown in Figure 3(a). When the PIN diodes are on, the gratings on the first layer are connected like a whole metal plate so that the incident V-pol EM wave is almost completely reflected. On the contrary, when the PIN diodes are off, the gratings work normally so that the incident V-pol EM wave almost completely goes through and transforms into its horizonal-polarization counterpart in a wideband. By setting periodical boundary conditions, the reflection and transmission coefficients of the meta-atom under V-pol incidence are shown in Figure 3(b). As is shown, in a wide frequency band, the difference between the reflection/transmission coefficients with diodes being on and off is greater than 10 dB. Specifically, the reflection of diodes being on is 9.1 dB higher than that with the diodes being off at 2.45 GHz, while the transmission with diodes being on is 11.6 dB lower than that with diodes being off.

To design an actual device, 10 × 10 meta-atoms with a total size of 200 mm × 200 mm are used for building a metasurface array. Photographs of the fabricated metasurface-based transmitter and its measurement setup in microwave anechoic chamber are shown in Figure S1 (Supplementary Material). Up to 200 PIN diodes have been welded on the first layer, which is fixed with other layers by plastic screws. To evaluate the far-field characteristics, the metasurface has been tested in a microwave anechoic chamber. Here, the metasurface sample is placed on a transmit table and a wideband horn is placed on another received table. By rotating the transmit table automatically, the scattering powers of all the angles are received by the horn antenna and recorded by a vector network analyzer (AV3672B).

To reduce the blockage of reflected signal, a log-periodic antenna with tiny cross-sectional area is utilized as an incident antenna. For V-pol incidence, we record V-pol reflection and H-pol transmission patterns, see Figure 3(c) and (d), respectively. Measured results of the metasurface transmitter manifest a good agreement with numerical ones at the condition of periodic boundaries. As shown in Figure 3(c), the measured V-pol reflection at direction of *θ* = 0° for all diodes being on is 8 dB higher than that for all diodes being off. For the measured H-pol transmission shown in Figure 3(d), the difference between the diodes being all on and all off is 11.7 dB at the direction of *θ* = 0°. Denote the condition of diodes being all on and all off as data 1 and 0 respectively, the receiver will easily decode 1 and 0 by comparing reflected or transmitted energy since satisfying differences are achieved between above two conditions.

Moreover, to increase the communication rate, high-order modulation such as four-level amplitude-shift-keying (ASK) can also be achieved by the metasurface-based transmitter. The strategy is to divide the metasurface into different areas, and then turn on PIN diodes in one area at a time to achieve multiple levels of reflection and transmission amplitudes. The voltage distribution for the cases of all on, all off, five rows on, eight rows on and two rows on are shown in Figure S2 (Supplementary Material). Corresponding measurement results are also compared in Figure 3(c) and (d). Regarding the reflection at direction of *θ* = 0°, the amplitudes for conditions of all on, eight rows on, five rows on, two rows on and all off are 0 dB, −2 dB, −4 dB, −5.9 dB, and −8dB, respectively. While regarding the transmission case at direction of *θ* = 0°, the amplitudes for those cases are −11.7 dB, −8.1 dB, −5 dB, −1.5 dB, and 0 dB, respectively. All above results indicate that multiple transmission and reflection intensity levels can be achieved which can be chosen to perform 4 ASK modulation.

## 4 Short- and long-distance experiment

**Figure 4:**
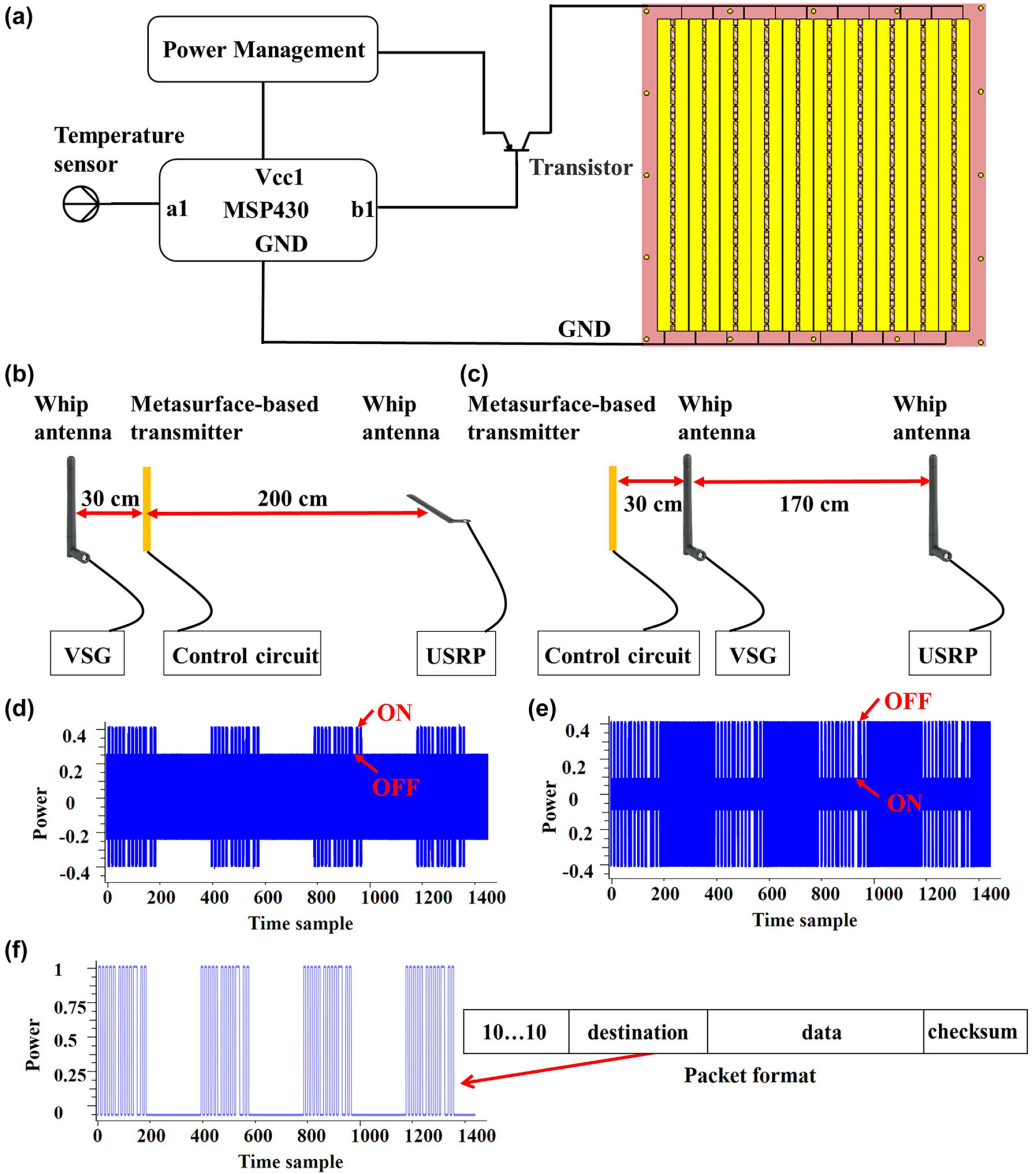
Short-distance experimental characterization of the metasurface-based front-back scattering communication system. (a) The control circuit of the metasurface-based transmitter. Measurement setup with well-marked distance relationships of the (b) reflection and (c) transmission channel. Initial signals received by the USRP of the (d) reflection channel and (e) transmission channel. (f) The decoded signal of both the reflection and transmission channels as well as the packet format.

To test the metasurface-based front-back scattering communication transmitter, a control circuit shown in Figure 4(a) has been designed. Here, the low-power MSP430 is utilized as a microcontroller which controls the voltages across diodes on metasurface by using a transistor to enhance the ability of the driven circuit. Moreover, the imposed voltages change according to the sensor data, which are data streams output via MSP430. To better emulate environmental signals as the carrier waves in our system, we use a RF vector signal generator (VSG) loaded with an omni-directional whip antenna, see measurement setup of reflection and transmission channels shown in Figure 4(b) and (c). In measurements, the universal software radio peripheral (USRP) loaded with an omni-directional whip antenna has been used as the receiver. For reflection channel, the whip antenna is placed vertically while it is placed horizontally for transmission channel due to polarization conversion of transmitted signal. The actual test environment and the schematic for the USRP receiver are shown in Figure S3 (Supplementary Material).

Figure 4(d) and (e) illustrate the initial signals received by the USRP in reflection and transmission channels when the power of signal generator is set to 0 dBm. To verify the optimization strategy of polarization conversion, we have made a detailed comparison between reflection and transmission channel. As illustrated in Figure 4(d) and (e), the signal-to-interference ratio for the reflection channel is much lower than that for the transmission channel. This is mainly because that the receiver obtains direct interference from ambient carrier wave source in reflection channel. Such a direct interference considerably limits the distance of BC, which is a bottleneck that needs to be addressed urgently. For transmission channel, we introduce polarization transformation by metasurface to eliminate the direct interference, which has been analyzed in detail in previous section.

Due to this difference, when output power of the generator is reduced, the bit error rate (BER) in reflection channel deteriorates faster than that in transmission case due to the influence of direct interference. When the power of signal generator is reduced to −35 dBm, the BER in reflection channel reaches 1 %. However, when the BER in transmission channel reaches 1 %, the power of the signal generator should be reduced to −45 dBm. Through the comparison of the transmission and reflection experiments, we conclude that the polarization transformation is a very effective way to solve the bottleneck of direct interference in BC.

Finally, the decoded signal as well as the packet format is shown in Figure 4(f). Although the reflected and transmitted 0 and 1 data streams of initial signals are complementary to each other, the temperature perceived by the sensor is identical. Therefore, the 0 and 1 data demodulated in transmission channel needs to be manually reversed relative to those in reflection channel. Then according to the package format and data encoding rules, the temperature will eventually be restored and displayed by the receiver.

**Figure 5:**
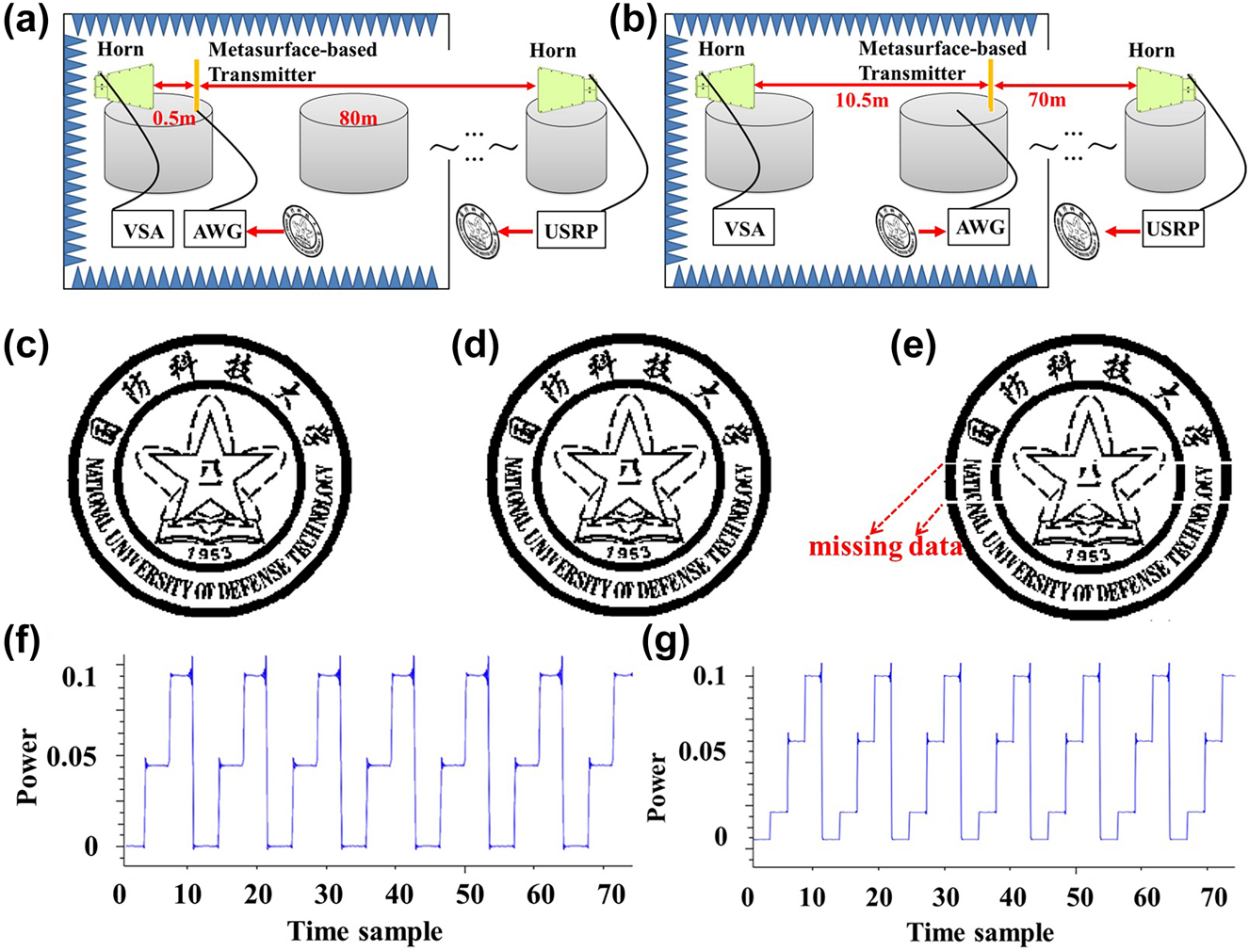
Long-distance data transmission test of the metasurface-based communication system. (a) Long-distance communication case 1: the distance from the generator antenna to the metasurface-based transmitter is 0.5 m, the distance from the metasurface-based transmitter to the receiver antenna is 80 m. (b) Long-distance communication case 2: the distance from the generator antenna to the metasurface-based transmitter is 10.5 m, the distance from the metasurface-based transmitter to the receiver antenna is 70 m. (c) The original picture transferred by the system. The received pictures for (d) case 1 and (e) case 2. The received signals for (f) 3ASK and (g) 4ASK.

To enhance the modulation rate of the metasurface-based transmitter, an arbitrary waveform generator (AWG) has been used as the controller to replace the initial control circuit, see Figure 5(a) and (b). Meanwhile, two long-distance communication cases are conceived. For case 1 shown in Figure 5(a), the distance from the generator antenna to the metasurface-based transmitter is set to be 0.5 m and that from the metasurface-based transmitter to the receiver antenna is fixed as 80 m.

While for case 2 shown in Figure 5(b), the distance from the generator antenna to the metasurface-based transmitter has been stretched to 10.5 m and then another distance is 70 m. Actual test environment is shown in Figure S4 (Supplementary Material). The original picture transferred by the system is shown in Figure 5(c). It is converted to 0 and 1 pixel-points in advance and stored in the AWG. Pictures received by the USRP for case 1 and case 2 are shown in Figure 5(d) and (e), respectively. In the actual measurement, the power of the RF generator is set to be 29 dBm and the modulation rate is 800 kbps. In this condition, the BERs of case 1 and case 2 are 0.014 % and 0.28 %, respectively. Due to the mentioned BERs, the picture has been almost perfectly reconstructed in case 1 while two thin lines are missed in the middle of received picture in case 2.

If the modulation rate is further increased, the BER will increase. This is mainly because that the response time of each PIN diode is difficult to maintain exactly the same. To further increase the communication rate, we can introduce multi-level ASK modulation to replace the initial OOK modulation. Here, we have illustrated that the metasurface-based transmitter can achieve multi-level transmission and reflection amplitudes by dividing the metasurface into different areas. In the test, two channels of the arbitrary waveform generator have been utilized to control two different areas of the metasurface. When the metasurface is split by half, three-level amplitudes can be achieved, see the received signals of USRP shown in Figure 5(f). While if the metasurface is split into two asymmetrical areas (one area has two rows of meta-atoms while the other owns eight rows), four-level amplitudes (4ASK modulation) were achieved as shown in Figure 5(g). In this condition, the communication rate will be doubled for the same modulation rate.

Although the experiment was conducted for cooperative carrier signals, our proposed metasurface-based front-back scattering communication can also be indeed extended to non-cooperative signals such as arbitrary indoor WiFi signals. Therein, a specificized receiver should be utilized by using a broadband amplitude detector. As the modulation is OOK, the received signal becomes a high- and low-level stream carrying the amplitude modulation information after passing through the broadband detector. In addition, since the power of real WiFi signal is fluctuated and the incident angle is uncertain, it will pose undesirable effect on the communication performance. Therefore, the transmitter and receiver should be further optimized to enable the system adaptive to the actual WiFi signals.

Lastly, we discuss the communication security of our proposed approach. To be honestly speaking, backscatter communication is a form of concealed communication in which the information is modulated secondarily in the signals already present in the environment. To further enhance the security, more complex waveforms need to be designed on the one hand. On the other hand, directional (point-to-point) communication with narrow beams can be realized by controlling the phase of each meta-atom in addition to current amplitude control.

## 5 Conclusions

To sum up, we have theoretically proposed and experimentally demonstrated a programmable amplitude-reconfigurable metasurface to achieve front-back scattering communication with high energy utilization efficiency. We first analyze the information modulation strategy of the metasurface-based front-back scattering communication transmitter and the polarization conversion technology to eliminate the direct interference. Then, a multi-layered meta-atom loaded with PIN diodes has been designed to achieve the reconfigurability of the reflection/transmission amplitude. For verification, a metasurface array of 10 × 10 meta-atoms with total size of 200 mm × 200 mm is utilized to build the transmitter. Numerical and experimental results verified the initial data can be finally restored and displayed by the receiver with satisfying BERs. At a modulation rate of 800 kbps and a distance of 80 m, the programmable metasurface-based front-back scattering communication system achieves distortion-free transmission of a picture. In addition, multiple amplitude shift keying modulation can also be realized by dividing the metasurface into different areas, which can further increase the communication rate. Our findings offer a general strategy to achieve high-efficiency binary or multi-binary amplitude modulation by using metasurface, which can be widely used in new generation of communication system, smart home, agriculture environmental monitoring, wearable information sensing and so on.

## Supplementary Material

Supplementary Material Details
