# Supplementary material for: Programmable metasurface for front-back scattering communication
Source: Nanophotonics. 2023 Aug 3;12(18):3653–61. doi: 10.1515/nanoph-2023-0365 (PMC11501942; doi:10.1515/nanoph-2023-0365)
Supplement: Supplementary file 1 — Supplementary Material Details [file j_nanoph-2023-0365_suppl_001.pdf]

Supplemental Material

Programable Metasurface for Front-back Scattering Communication

Haipeng Li,[1] Kewei Xin,[2†] Haiyang Ding,[2*] Tangjing Li[3], Guangwei Hu[4*], He-Xiu Xu[3*]
[1]National University of Defense Technology, Test Center, Xi'an, China, 710106
[2]National University of Defense Technology, College of Information and Communication, Wuhan, China, 430035
[3]Air force Engineering University, Air and Missile Defense College, Wuhan, China, 710051
[4]Nanyang Technological University, School of Electrical and Electronic Engineering, Singapore, 637371

1. Measurement setup of the metasurface

2. Voltage distribution for generating multiple amplitudes

3. Actual test setup for the short-distance experiment

4. Actual test setup for the long-distance experiment

1. Measurement setup of the metasurface

To design an actual metasurface-based front-back scattering communication transmitter, as shown in Figure S1, 10×10 elements with a total size of 200mm× 200mm are used for building a metasurface array. Two hundred of PIN diodes have been welded on the first layer, which is fixed with other layers by plastic screws. To evaluate the far-field characteristics, the metasurface has been tested in a microwave anechoic chamber. The schematic diagram of the measurement is also shown in Fig. S1.

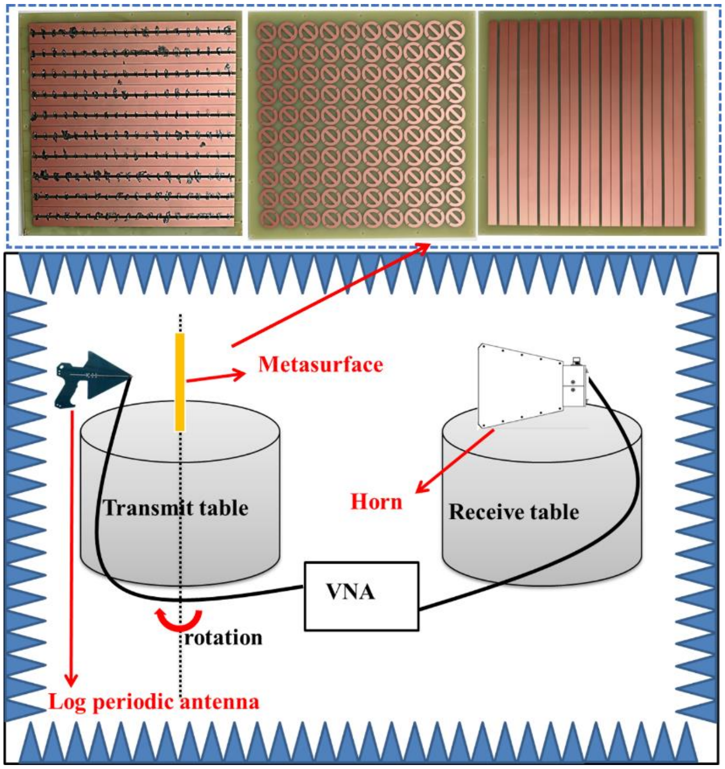

FIGURE S1: Photograph of the fabricated metasurface-based transmitter and its measurement setup in microwave anechoic chamber.

2. Voltage distributions for generating multiple amplitudes

To increase the communication rate, high-order modulation such as four-level ASK can also be achieved by the metasurface-based transmitter. The strategy is to divide the metasurface into

different areas, and then make PIN diodes turn on in one area at a time to achieve multiple levels of reflection and transmission amplitudes. The voltage distributions for the cases of all on, all off, five rows on, eight rows on and two rows on are shown in Table S1 and the number of the row on the metasurface is shown in Figure. S2.

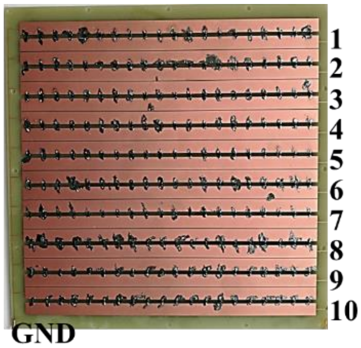

FIGURE S2: The number of the row on the metasurface.

Table S1
Voltage distributions for generating multiple amplitudes.

	1	2	3	4	5	6	7	8	9	10
All on	2V	2V	2V	2V	2V	2V	2V	2V	2V	2V
All off	0	0	0	0	0	0	0	0	0	0
Five rows on	2V	2V	2V	2V	2V	0	0	0	0	0
Eight rows on	2V	2V	2V	2V	2V	2V	2V	2V	0	0
Two rows on	2V	2V	0	0	0	0	0	0	0	0

3. Actual test setup for short-distance experiments

In actual test, as shown in Figure S3, a universal software radio peripheral (USRP) loaded with an omni-directional whip antenna has been used as the receiver. For reflection channel, the whip antenna is placed vertically while it is placed horizontally for transmission channel due to polarization conversion of transmitted signal. For the USRP receiver, the signal received by the whip antenna is firstly sampled at zero intermediate frequency (IF), and then it goes through a threshold judgment and finally is displayed on an oscilloscope.

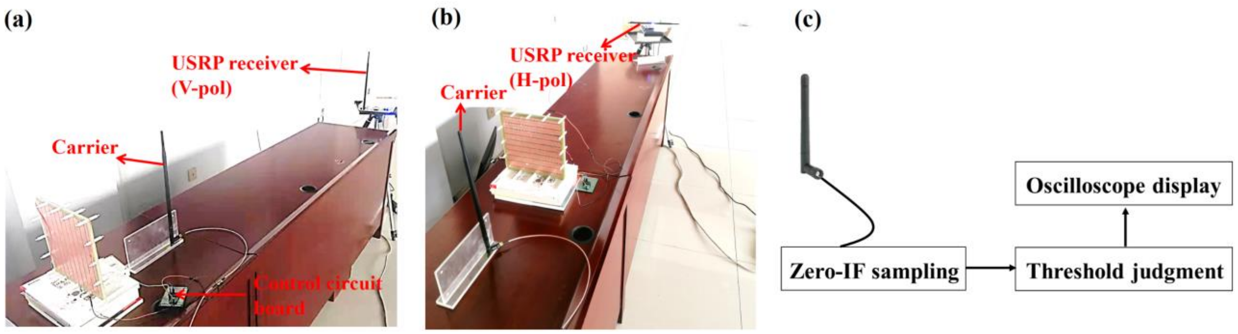

FIGURE S3: Actual test environment and the receiver schematic for the short-distance experiment. Actual test environment for (a) reflection and (b) transmission channel. (c) The schematic for the USRP receiver.

4. Actual test setup for long-distance experiments

In actual test, as shown in Figure S4, the metasurface-based transmitter as well as the carrier wave source are placed in the microwave anechoic chamber, while the USRP-based receiver is placed on the outdoor rooftop to extend the communication distance. For case 1, the RF source is placed near the metasurface-based transmitter. For case 2, the RF source is arranged far from the metasurface-based transmitter.

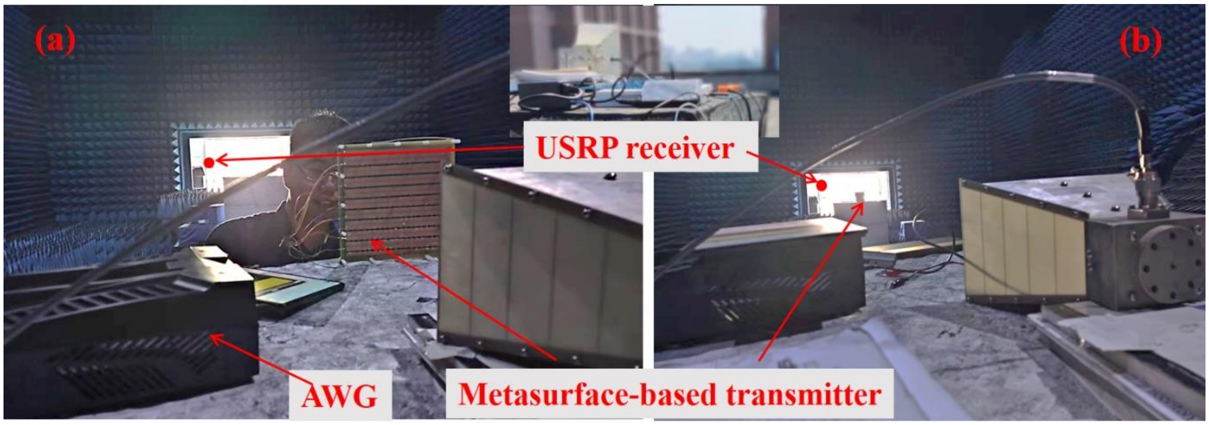

FIGURE S4: Actual test setup for the long-distance experiment. Actual test environment for (a) case 1 and (b) case 2.
